# Sociotechnical imaginaries and public communication: Analytical framework and empirical illustration using the case of artificial intelligence

**DOI:** 10.1177/13548565251338192

**Published:** 2025-04-28

**Authors:** Saba Rebecca Brause, Mike S Schäfer, Christian Katzenbach, Yishu Mao, Vanessa Richter, Jing Zeng

**Affiliations:** 27217 University of Zurich, Switzerland; 9168 University of Bremen, Germany; 473924 Alexander von Humboldt Institute for Internet and Society, Germany; 28263 Max Planck Institute for the History of Science, Germany; 9168 University of Bremen, Germany; 27217 University of Zurich, Switzerland

**Keywords:** Sociotechnical imaginaries, public communication of science and technology, artificial intelligence, scientific public sphere, social construction of technologies

## Abstract

The concept of sociotechnical imaginaries (SIs) has been widely used and proven fruitful to understand diverging trajectories of technologies. While scholars have acknowledged the multi-layered materialisation of SIs and highlighted the importance of the communicative layer therein, this aspect has remained under-conceptualised. Therefore, we propose an analytical framework to better understand Sociotechnical Imaginaries in Public Communication (SIPCs), defined as *publicly* constructed visions of (un)desirable sociotechnical futures that guide action, mobilise resources and lay out trajectories for the materialisation or prevention of those futures. In this article, we first discuss relevant strands of research on SIs and public communication. We then lay out the analytical framework of SIPCs that enables the rigorous reconstruction and comparison of sociotechnical imaginaries in public and mediated communication. Finally, we illustrate the framework with examples from public communication about artificial intelligence, an emerging key technology in contemporary societies.

## Introduction

Public communication about technologies – that is, communication that is in principle open to a broad, diverse audience and distributed via technical means such as news and social media (Brossard, 2013; McQuail, 2010) in fictional or non-fictional form, audiovisual, text or other formats – does not merely disseminate knowledge (Bucchi and Trench, 2021; Strekalova, 2015). It also contributes to the communicative construction of these technologies and their future role in societies (Pentzold et al., 2020). In doing so, it shapes sociotechnical imaginaries.

The concept of sociotechnical imaginaries (SIs, Jasanoff, 2015a) has been widely used in Science and Technology Studies (STS). The concept has laid out how imaginaries are formed around technologies, influenced by both societal and technological factors, how they can manifest in different realms of life, and how they can impact the chances, challenges and developmental trajectories of technologies. But while the role of public communication for the construction of SIs has been recognised (Jasanoff, 2015a, 2016), it remains under-conceptualised.

This paper adds to scholarship on SIs by sharpening and illustrating the role of public communication in this concept. Therefore, we propose an analytical framework – Sociotechnical Imaginaries in Public Communication (SIPCs) – that adds elements from scholarship on public communication about technologies (PCT) to the SI concept and might prove useful for future research.

We understand SIPCs as *publicly* constructed visions of (un)desirable sociotechnical futures. They can guide action, mobilise resources and lay out trajectories for the materialisation or prevention of those futures. As such, they differ in several respects from other concepts developed for analysing communication, like frames, narratives or discourse, as they draw from and expand on SIs research, inheriting several characteristics that set them apart: SIPCs are explicitly *future-oriented*, as they project pathways for how a society should (or should not) use and evolve with technologies (Jasanoff, 2015a; Jasanoff and Kim, 2009). They are also aimed at inspiring *collective* support or opposition to make them *actionable* (Beck et al., 2021; Kuchler and Stigson, 2024). Hence, they are connected to concrete *actions* for shaping those futures and are therefore not merely descriptive but also *prescriptive*. If they attract enough support among stakeholders and members of the public, they can contribute to the realisation or prevention of the envisioned futures (Jasanoff, 2015a, 2015b; Jasanoff and Kim, 2009). Finally, they are *materially embedded*, as they are both shaping and being shaped by the materiality of technologies and infrastructures that they evolve in (Jasanoff, 2015b; Smith and Tidwell, 2016). These distinctive characteristics have made SIs a widely used concept in analyses of sociotechnical developments and visions. Yet, while this research often relies on and analyses mediated, public communication, the operationalisation of SIs in public communication has remained underdeveloped.

We aim to close this gap and propose an analytical framework to do so – not aiming to replace SIs as a concept, but to *explicate its inherent communicative layer* in order to enable more systematic, rigorous analyses of public and mediated representations of SIs in the future. To do so, we first engage with the SI concept and highlight the need for further specification of the role of public communication with the help of scholarship on public communication about technologies (PCT). Second, we lay out a multidimensional analytical framework for capturing public imaginaries of technology analytically and empirically. Third, we then illustrate this framework with examples of public communication about artificial intelligence, an emerging key technology in contemporary societies (Stone et al., 2016), which is widely discussed in news and social media (Brennen et al., 2019; Zeng et al., 2022) and whose role in society is still being negotiated (Jobin and Katzenbach, 2023).

## Sociotechnical imaginaries and public communication about technologies: Relevant strands of research

### Sociotechnical imaginaries of technologies

Social scientists have long used the concept of imaginaries to analyse collective constructions of social realities, such as national identities, modernity or globalisation (Anderson, 1983; Appadurai, 1996; Taylor, 2004). Focussing on the emergence, characteristics and implications of technologies, STS scholars developed the concept of SIs, defined as ‘collectively held, institutionally stabilised, and publicly performed visions of desirable futures, animated by shared understandings of forms of social life and social order attainable through, and supportive of, advances in science and technology’ (Jasanoff, 2015a: 4). SIs are context-specific – ‘culturally particular’, ‘temporally situated’, materially bound and spatially anchored (Jasanoff, 2015a: 19) – and can therefore elucidate locally varied and temporally specific understandings and, by extension, developments of technologies (Burri, 2015; Jasanoff and Kim, 2009; Miller, 2015).

The framework of SIs has been widely adopted in scholarship and possesses pronounced strengths: First, it underlines that technological development does not follow techno-deterministic paths; instead, it is co-produced by sociopolitical or sociocultural factors like social values, power relations, political regulation or public perceptions. Second, it is a multifaceted concept which captures various layers of the mutual shaping of society and technology (Richter et al., 2023). Third, it is oriented toward the future and lends itself well to prognoses about technological development. Fourth, it provides a conceptual and analytical framework for examining how *different* sociopolitical and sociocultural contexts produce different – and potentially *competing* – imaginaries that may favour, and eventually result in, different developmental pathways for technologies (Mager and Katzenbach, 2021).

Over time, the concept has seen several expansions: First, scholars acknowledged that diverse actors can play a role in shaping SIs. While the initial conceptualisation by Jasanoff and Kim (2009) focused largely on the role of nation-states, Jasanoff (2015a) later expanded the framework to acknowledge the impact of other stakeholders such as corporations (Olbrich and Witjes, 2016; Smith, 2015), supranational organisations like the European Commission and Parliament (Rieder, 2018; Wijermars and Makhortykh, 2022) or other organisations or collective actors like higher education institutions, professional societies, expert groups, non-governmental organisations or (new) social movements (Jasanoff, 2015a; Olbrich and Witjes, 2016).

Second, scholars acknowledged that while different stakeholders may be competing to establish *dominant* SIs, multiple and potentially competing imaginaries can *co-exist* at the same time within and between different societal forums and levels – from local communities over regions and nations to the transnational and global level (Jasanoff, 2015b; Mager and Katzenbach, 2021). In fact, this co-existence is essential to the discursive negotiation that SIs undergo in their institutionalisation and reflects the potential for changes in dominant SIs as the discourse around a technology shifts or changes paradigms, as seen, for example, with nuclear energy (Jasanoff and Kim, 2009).

Notably, however, empirical research has not been in line with these conceptual developments. Empirical studies have largely focused on dominant SIs (or pervasive counter-imaginaries) proposed by a small set of pre-defined stakeholders, analysing their policy or strategy documents, advertisements or other direct communication, sometimes complemented by interviews or observational methods (e.g. Burri, 2015; Felt and Öchsner, 2019; Mager, 2023; Sadowski and Bendor, 2019). This approach risks leaving SIs unacknowledged that could co-exist at a different societal level or be promoted by previously unidentified stakeholders or groups. Moreover, it might produce blindspots regarding stakeholders which can shape dominant SIs or counter-imaginaries in more subtle ways. It is also not well suited to public, mediated communication in legacy or social media where different stakeholders exchange views that contribute to the construction of imaginaries.

### Sociotechnical imaginaries and public communication

Against this backdrop, we argue that the role of public communication in SIs is still underdeveloped. Public communication has been conceptualised as communication that is distributed via technical means and is, in principle, open to a broad and diverse audience (McQuail 2010). It serves to inform citizens and stakeholders alike about societal developments – such as the emergence of new technologies – and allows for opinion formation and participation in broader societal debates about desirable futures and directions to get there (Eisenegger and Schäfer 2023). Accordingly, public communication is seen as highly important in contemporary societies and its underlying values and norms have been widely discussed (e.g. Ferree et al., 2002).

Public communication, understood like that, includes news coverage and social media, fictional or non-fictional communication, and communication in textual, audiovisual or other formats (Dudo, 2015) – and SI scholarship has highlighted its importance for the emergence and manifestation of SIs. For instance, Jasanoff posits that SIs need to be ‘publicly performed’, meaning they are ‘accompanied by concrete actions that have the power to satisfy supporters and persuade new adherents’ (Jasanoff, 2016: 83). While this performance of SIs can take various forms (Rudek, 2022), one crucial aspect is that imaginaries need to be *expressed* in public, that is, they find ‘expression in the mass media’, ‘policy documents’, and other forms of public communication (Jasanoff, 2015a: 27). Once public, they can garner collective support and supporters. Put differently, public exposure and promotion is necessary to move mere narratives around technologies towards shared future visions that are publicly supported through social, economic, or political collective action. If successful, the public communication of SIs has a powerful performative function: it can ‘coproduce the installment of these futures’ (Bareis and Katzenbach, 2022: 17) by mobilising stakeholders to support and endow the respective visions with resources and investments.

But the role of public communication in the formation and institutionalisation of SIs has remained under-conceptualised so far in SIs scholarship, which has resulted in a variegated and often incomplete operationalisation of public and mediated representations of SIs: First, studies on the communicative layer of SIs are rare in general. Second, existing scholarship has largely refrained from systematically laying out and operationalising SIs in public communication. Instead, it has used the framework as an overarching or ‘sensitising’ concept to analyse discourse, themes and narratives (Leder Mackley and Jewitt, 2022; Scott Hansen, 2022; Tutton, 2021; Wang et al., 2023) or mentioned SIs as a conceptual reference while working analytically with different concepts such as ‘key frames’ (Eaton et al., 2014), ‘media frames’ (Vicente and Dias-Trindade, 2021) and ‘promissory themes’ (Lupton, 2017). Third, some studies have used SIs as essentially unidimensional. Neresini et al. (2020), for example, conceptualised ‘sociotechnical future scenarios’ (FS) as ‘discursive frames about a specific situation that are promoted and constructed through media representation’ that might ‘create and shape expectations or … prefigure strategies of action’ (2023) (148). When assessing FS of energy transition in news coverage, they distinguish different stakeholders, but on a *content*-level, they treat SIs as unidimensional ‘topics’, such as ‘national policies for decreasing energy waste’ despite the multifaceted nature of the framework. Fourth, some research has captured different content-layers of SIs, but treated the potentially diverse *stakeholders* in the public arena as a monolithic entity instead of a set of speakers with different positions, strategic aims and messages. Upham et al. (2023), in their study of international news imaginaries of Norwegian and Icelandic data centres, analysed ‘international news’ as a single entity, omitting the potential multiplicity of stakeholders that may promote or shape SIs *within* these media. Fifth, some studies have omitted relevant dimensions of SIs in their analyses. Examining virtual reality-related SIs in fiction and nonfiction media, Preece et al. (2022) differentiated speakers, temporality and geography of SIs, different technologies, associated objective and dominant tropes, and text format and language norms. But they did not operationalise the desirability and action-oriented dimensions of SIs, despite their centrality to the concept.

In sum, a more elaborate explication of SIs’ communicative dimension is lacking, even though it has been acknowledged as an important facet of SIs. As such, our argument also resonates with STS scholars’ criticisms regarding a lack of rigorous methodologies in STS for analysing complex sociotechnical concepts, particularly for larger-scale, quantitative analyses (Hacket et al., 2007; Wyatt et al., 2017).

Therefore, we propose to specify *Sociotechnical Imaginaries in Public Communication (SIPCs)* adopting four core aspects from the SI concept – the existence of a future-oriented *vision*, the specific *technology* in the vision, the *desirability* assessment of the vision, and the *spatio-temporal* situation of the vision – and supplementing them with insights from scholarship on PCT (see next section).

### Scholarship on public communication about technologies

Scholarship on PCT is manifold. Adopting the understanding of public communication outlined above (McQuail 2010) and focussing on technologies such as nuclear (e.g. Gamson and Modigliani, 1989), bio- (e.g. Durant et al., 1998) and nanotechnology (e.g. Metag and Marcinkowski, 2014), scholars from various social sciences have analysed PCT (for an overview, see McCluskey et al., 2016). Similar to scholars of SIs, their interest was often driven by the recognition that public debates are important influences on the implementation, public acceptance and development of technologies (see Bucchi and Trench, 2021). Scholars often focused on mediated public communication; early on especially on news media coverage about technologies (e.g. Eyck, 2005; Stephens et al., 2009), in recent years also on online and social media (e.g. Cacciatore et al., 2012; Veltri, 2013).

PCT scholarship can contribute to the understanding of *SIPCs* in two ways. Fundamentally, it underlines the importance of public and particularly *mediated* public communication about technologies that is already implied in the SI concept: News media, but also social media have high societal visibility and wide reach among citizens, stakeholders and decision-makers when it comes to technologies (e.g. National Science Board, 2018). Consequently, mediated portrayals of technologies can profoundly influence the trajectories of technologies (Brossard, 2013; Goggin, 2012), put them on public and policy agendas, highlight their benefits and risks, and frame them in ways that influence regulation and acceptance (e.g. Edler and James, 2015; Strekalova, 2015).

Furthermore, and more specifically, approaches developed and applied in scholarship on PCT can also be utilised for explicating SIPCs. Several of them originate from public sphere, differentiation and general systems theory and fundamentally understand public communication as a competition between different speakers and discursive positions for public visibility and, ultimately, hegemony (Ferree et al., 2002; Gerhards and Schäfer, 2006, 2010; Hilgartner and Bosk, 1988). Given the importance of public communication in society and the central role of news and social media therein, these approaches assume that individual and organisational actors strive to position themselves prominently in public, and aim to promote their perspectives, evaluations and recommendations for action. Accordingly, scholars have looked at two dimensions when analysing public communication about technologies (e.g. Ferree et al., 2002; Gerhards et al., 1998): the *speakers* in public communication and their relative success in positioning themselves prominently in public on a given topic (Ferree et al., 2002; Gerhards and Schäfer, 2009), and the *content* of public communication, that is, how prominently a technology is presented and how it is framed in public discussion. Framing, in this tradition, is understood as ‘select[ing] some aspects of a perceived reality and mak[ing] them more salient in a communicating text’ (Entman, 1993: 52; Matthes, 2009), thus guiding public interpretation of an issue. According to Entman (Entman, 1993: 52f), such frames may include different definitions of the object in question (‘problem definitions’), its origins (‘causal interpretations’) as well as ‘moral evaluations’ and ‘treatment recommendations’.

These approaches mesh well with the SIs concept – for example, by emphasising the importance of ‘moral evaluations’ and the ‘desirability’ of a given technology. However, they also underscore less formalised aspects of SIs, particularly the *speaker* dimension, which has widely been overlooked in scholarship on the communicative layer of SIs, and which formalises the potential competition between different speakers and, ultimately, imaginaries in the public sphere, but also the need for mediated calls for action, such as formalised in *treatment recommendations*, which are implied in SIs but often not explicated. We will draw from these aspects of PCT for further explicating SIPCs. Importantly, our proposal does not aim to replace rhetorical, narrative or discourse analysis, rather, it is intended to offer a more refined tool for analyses of SIs, which rely on the distinctive future-oriented, collective, actionable, and material characteristics of the concept, but empirically focus on their communicative layer.

## How to understand and assess sociotechnical imaginaries in public communication: Proposing an analytical framework

Scholarship on both SIs and on PCT emphasises the importance of social perceptions of technologies and the formative power of public and mediated communication on developmental paths of technologies. As this aspect has been less pronounced in SI scholarship so far, however, we propose the notion of *Sociotechnical Imaginaries in Public Communication (SIPCs)*, which combines elements from SIs and PCT scholarship. SIPCs can be understood as *publicly constructed visions of (un)desirable sociotechnical futures that guide action, mobilise resources and lay out trajectories for the materialisation or prevention of those futures*. Herein, we understand public construction as a communicative process that can take place in a variety of public fora, including legacy news media, digital-born news media as well as social media, and in traditional corporate and institutional communication channels, such as advertising, marketing, PR or governmental and stakeholder reports, among others (Gerhards and Schäfer, 2006, 2010; Hilgartner and Bosk, 1988). It can take place in non-fictional and fictional form and in different communicative modalities like text, sound and audiovisual content.

Correspondingly, and in line with SI and PCT scholarship, we also assume that different, even contradictory SIPCs regarding the same technology may co-exist, that they may be propagated by different stakeholders, that they may translate into different assumptions about potential actions, and that they may compete for collective support. Furthermore, imaginaries may focus on entire technologies, but also on specific facets or applications of those; in other words: they may centre different variants of technological objects.

**Figure 1.**
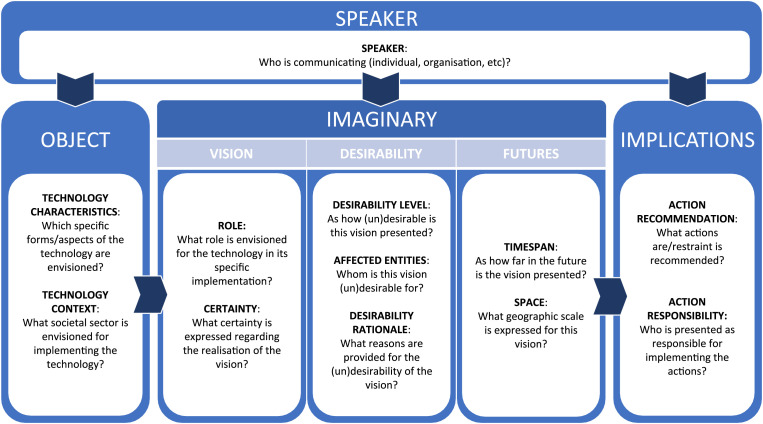
Constitutive dimensions of sociotechnical imaginaries in public communication.

Based on these assumptions, we suggest to assess SIPCs as encompassing six dimensions and up to 12 elements that characterise how visions of (un)desirable sociotechnical futures manifest in public communication (see also Figure 1).

### The core of sociotechnical imaginaries in public communication: Vision, desirability, and futures regarding a technological object

Scholarship on SIs (Jasanoff, 2015a) can be used to explicate and structure the **core dimensions of SIPCs** as they manifest in public communication: imaginaries contain (1) a *vision for a technology’s future role* in society and a (2) *desirability* evaluation of the vision. They may also contain a (3) *spatio-temporal* specification of the vision for the *future*, and they are always attached to a specific (4) technological *object*. Below, we elaborate on these four dimensions encompassing nine elements of the analytical framework.

• The first dimension is the **vision** relating to the technology. This dimension captures the meaning and potential attached to a technology for the future of a society. We suggest that it contains two elements. The primary element within the vision is the *role* that the technology is envisioned to assume in a given society. It specifies the goal pursued with a technology as it is being developed and implemented. For instance, a technology can be presented as a means to increase productivity and efficiency, to facilitate surveillance, or to reduce accidents. A second element are expressions of *(un)certainty* about the vision – that is, as how certain it is presented that a given vision can be realised.
• The second dimension adds an assessment of the **desirability** or undesirability to the vision. This dimension captures the normative specification of the vision. We propose that this involves three elements, namely the *level of desirability*, the *affected entities*, for example, whom it might be (un)desirable for, and the *rationale* for the (un)desirability. In some cases, the desirability evaluation might be neither positive, nor negative, but remain neutral or ambiguous. *Affected entities* could include, for instance, certain groups of people, societal realms like the economy or science, but also animals, or broad systems like the earth’s climate. (*Un)desirability rationales* provide the reasoning for why the vision is presented as (un)desirable. For instance, the envisioned role for the technology in society may harm parts of society, or other entities, such as animals or the environment; or it may improve outcomes, for example, for health, education or the economy.
• The third dimension concerns the specific **spatio-temporal ‘future’** for which a vision is proposed, that is, its specification in *time* and *space.* The former specifies the timespan envisioned for the realisation of the vision. The latter sets out the envisioned space where the vision is considered to be realised. This could, for instance, be a local community, a region, a nation state or the entire globe.
• The technological **object** of the imaginary captures the variety of technologies or parts thereof, that can be referred to by an imaginary. We suggest that it contains one element regarding the technology’s characteristics, and another concerning the context of the technology’s application. *Technology characteristics* cover the specific forms or aspects of the technology that the vision refers to, such as large language models, humanoid robots, or voice assistants in the case of AI. The *technology context* refers to the sector of envisioned application or implementation, including, for instance, the home, the military, or healthcare sector.

### The contexts of sociotechnical imaginaries in public communication: Speakers & implications

To **contextualise these imaginaries**, we first include the speaker, that is, the *individual or collective stakeholder expressing an imaginary,* as proposed by PCT literature (Ferree et al., 2002; Gerhards and Schäfer, 2009). Furthermore, we draw from the framing framework laid out above (Entman, 1993; Matthes, 2009), particularly its ‘treatment recommendation’, to formalise the *implications of SIPCs*.

• The **speaker** denotes the person or stakeholder formulating and promoting a vision of a technology, and their characteristics (e.g. their profession, nationality, etc). Speakers can be individuals, but also groups of people (like social movements) or organisations (like corporations or a university) as long as they present themselves as speakers with a single voice and reflect on any element of the SIPC in question. If speakers reference other speakers’ reflections on SIPC elements (see e.g. Koven, 2002), pure reproduction should be considered as the voice of the quoted speaker, whereas comments, extensions or evaluations of those quotes by the speaker should be part of their own voice. While we recognise that non-human actants like regulations or communicative technologies can also carry a voice and shape SIPCs (see also Cooren, 2010), we suggest that speaker analysis should in such cases focus on the human authors or creators of such objects. The ‘speaker’ dimension does not contain several elements, therefore, the dimension can be considered to be an element in and of itself.
• The **implications of the vision** formalise the action-oriented side of imaginaries. This dimension involves both *recommendations for action*, and a specification of the *actors deemed responsible* for making the vision a reality. These recommendations can support specific actions or suggest *not* to act in a certain way.

The SIPCs framework lends itself to the study of different forms of public communication – from legacy to digital media, from news to social media, in fictional or non-fictional form, in different social, political and cultural contexts, in different modalities, and referring to different technologies. It should also enable both qualitative and quantitative analyses of imaginaries. So far, studies focussing on imaginaries have largely used qualitative, ethnographic and discourse-analytical methodology (cf. Richter et al., 2023). The SIPCs framework still allows for such in-depth case studies while also enabling larger-scale and comparative analyses of public communication.

## Empirical illustration: Public imaginaries of AI in U.S. news coverage

To illustrate how the framework can support the study of sociotechnical imaginaries in public communication, we provide examples of SIPCs of artificial intelligence (AI) in this section, laying out similarities and differences across the individual dimensions of the framework.

AI is an instructive test case for several reasons: First, AI has been widely claimed to transform society, economy and science itself (Bughin et al., 2018; Stone et al., 2016; The Alan Turing Institute and The Royal Society, 2019). Furthermore, it has been extensively discussed in both news and social media, especially since ChatGPTs publication in late 2022, represented in both fictional and non-fictional form, and interpreted in both strongly positive and negative ways (Brennen et al., 2019; Elyamany, 2023; Zeng et al., 2022). AI also lends itself to testing the framework because it is a complex and evolving technology whose shape, role and place in society are still being negotiated (Natale and Ballatore, 2020). Moreover, while research has attempted to identify common traits in AI definitions – highlighting perceptual, information-processing, decision-making and goal-achieving characteristics (Samoili et al., 2021), a final conceptualisation remains outstanding (Jobin and Katzenbach, 2023). This interpretive flexibility (Bijker, 2009) engenders different understandings of what AI *is* or *ought to be*, and produces hopes and fears for its future development and implementation (Cave and Dihal, 2019).

We illustrate AI-related SIPCs by drawing from U.S. news coverage between 2012 and 2021. Through our discussion and comparison of three different example imaginaries in public communication in a single type of medium, within a single national context, and within a set timespan, we aim to show the potential co-existence of, and competition between imaginaries related to an emerging technology, as its boundaries, role and place in society, and implications for action are still being negotiated.

Examples were drawn from a larger analysis of print and online articles from *The New York Times*, *The Washington Post*, *Wall Street Journal*, *USA Today*, and the *New York Post*, and acquired through a Factiva database search for mentions of ‘artificial intelligence’ in the title or leading paragraph, between 01 January 2012 and 01 July 2021, and in English. To reduce the sample for in-depth analysis while ensuring topical variety, the preliminary corpus of 2.370^1^ articles was pre-filtered through Latent Dirichlet Allocation-based topic modelling (Blei et al., 2003). Topics were then regrouped into five larger ‘imaginary contexts’ consisting of: Economy & Business, Research & Education, Culture & Media, Healthcare, and Risk & Governance. Articles were equally sampled from each topic within each ‘imaginary context’ conditionally of a minimum keyword count of five mentions of ‘artificial intelligence’ or ‘AI’ in the text body to ensure strong article focus on AI, and of a minimum 50% topic fit to ensure clear topical boundaries. A thematic analysis of the articles used the proposed framework’s six dimensions and 12 constitutive elements as ‘analytical objectives’ (Guest et al., 2012). For inclusion, each article was first scanned for markers of future-orientation, such as ‘will’, ‘could’, or ‘in the future’, then checked for the description of at least one envisioned ‘role’ for AI in the article. Articles were sampled until themes for the ‘role’ element reached saturation, or down to 50% topic fit. Articles were regrouped according to emerging ‘role’ themes and thematic analysis was then performed for the remaining conceptual elements. Three of the resulting imaginaries were chosen as illustrations for this methodologically focused article because they displayed both similarities and differences across the different elements of the framework.

In this article, and in line with the qualitative analytical approach for these example illustrations, we do not analyse overall strength of the imaginaries, but focus instead on their shape, as well as similarities and differences across the framework’s elements and dimensions. We leave further validation as well as large-scale quantitative analyses to further research.

These examples differ strongly across the role and object elements, as well as the desirability and implications dimension, while sharing certain similarities across the futures dimension, the certainty element, and the speaker dimension (see Table 1).

**Table 1.** Example imaginaries: (1) Fighting crime, (2) Enhancing everyday life and (3) Manipulating public perceptions.

Element	(1) Fighting crime	(2) Enhancing everyday life	(3) Manipulating public perceptions
Speakers	- Technology sector: CEO & senior employees - Civil society: experts, activists, rights group members - Government: representatives & officials	- Technology sector: executives, senior employees, organisations - Civil society: university professor, consumer survey, journalist	- Technology sector: organisations & founders - Civil society: university professors
Technology characteristics	- Facial-Recognition Technologies - AI/artificial intelligence	- Virtual assistants - Smart connected devices - AI/artificial intelligence	- Deepfakes - AI/artificial intelligence
Technology context	- Law enforcement & national security - Social Media	- Home - Everyday life - Economic sectors (mobility, consumer technology)	- Social Media
Role	HELPING FIGHT CRIME (a) by monitoring crowds (b) by monitoring & identifying crime online	ENHANCING EVERYDAY LIFE FOR CONSUMERS (a) by automating the home & everyday life (b) by facilitating entertainment activities (c) by improving existing consumer technologies	MANIPULATING PUBLIC PERCEPTIONS (a) by spreading distrust (b) by altering videos
Certainty	- No clear theme (from unspecified over uncertain to certain)	- Elevated	- Elevated
Desirability evaluation	- Desirable - Ambiguous	- Ambiguous	- Undesirable
Affected entities	(+) Government services, Facebook, crime victims (+/−) People	(+/−) Consumers	(−) Public debate, democracy, the public, politicians
Desirability rationale	For government services, Facebook, crime victims: (+) facilitating the fight against crime For people: (+) preventing crime & keeping people safe (−) risk of surveillance/privacy invasion (−) risk of bias & inaccuracies	For consumers: (+) easier control of home appliances (+) convenience (+) higher sense of security for voice data (+) easier contact with loved ones (−) risk of technological lock-in (−) loss of ability to think	For public debate, democracy, politicians: (−) spreading distrust (−) eroding public debate For the public: (−) increasingly difficult detection of misinformation
Timespan	- Not specified	- No clear theme (from ‘soon’ and ‘this fall’ to ‘in the coming years’)	- Already happening, but getting worse
Space	- No clear theme (including USA, China, EU, France)	- Not specified	- Not specified
Action recommendation	For regulators - Regulating use of facial-recognition technology	Next steps for tech companies: - developing & rolling out news features - collaborating with other companies	For policymakers: - Limit deepfake spread - Boost consumer trust in quality news For policymakers, citizens, social media platforms - Be prepared for & aware of deepfake spread
Action responsibility	- Regulators	- Technology companies	- Policymakers - Citizens - Social media platforms

First, regarding differences, the three imaginaries represent different envisioned roles and specific types of AI. In the first imaginary, AI technologies, such as facial recognition, are envisaged to help fight crime, both online and offline. For example, one article titled that ‘Facebook taps artificial intelligence in new push to block terrorist propaganda’ (Guynn, 2017) and two guest authors from Clearview AI argued that ‘Responsible private companies… should be permitted to access facial-recognition technologies to prevent identity theft and otherwise deter criminality’ (Abrams and Wolosky, 2021). In contrast, the second imaginary envisions virtual assistants, connected devices and ‘AI’ to enhance everyday life, for instance, by automating people’s homes: ‘But you’re not buying just a talking jukebox. Alexa, Siri and Google Assistant also want to adjust the thermostat, fill your picture frame or even microwave your popcorn’ (Fowler, 2018). The third imaginary posits that deepfakes and ‘AI’ will manipulate public perceptions through their potential use for altering videos and spreading distrust. For instance, the director of a research centre ‘expects “cheap fakes” and the more sophisticated deepfakes to proliferate ahead of the presidential election as part of shadowy campaigns to sway public opinion’ (Guynn, 2020).

The three imaginaries also differ in their desirability assessment, expected affected entities and desirability rationales. Indeed, the first, crime-fighting imaginary was described as desirable for several entities, including government services (e.g. Harwell and Dou, 2020) and potential victims of crimes (e.g. Abrams and Wolosky, 2021) because it would make the fight against crime easier: it ‘would help to identify perpetrators of crime as well as victims of abuse, and to apprehend terrorists, both foreign and domestic’ (Abrams and Wolosky, 2021). However, ambiguity was expressed for people because it could both ‘keep people safe’ (Harwell and Dou, 2020), as well as veer into ‘mass surveillance’ (Meichtry and Schechner, 2021), for example. The second imaginary was described as ambiguous for consumers. Arguments ranged, for instance, from easier control of home appliances (‘Now with a speaker and the right connected gizmo, you can walk into a room and turn on the lights without touching a button. Or control the TV without a remote. Amazon even sells an Alexa-operated microwave that cooks, tracks and reorders popcorn’ (Fowler, 2018)) to fears of intellectual laziness risks of an ‘AI-driven rise in “mental obesity”’ (Baig, 2018). The third imaginary was considered undesirable for politicians (Guynn, 2020) and the public (Chiu, 2019), but also non-human constructs such as public debate and democracy (Vaccari and Chadwick, 2020). This was founded in expectations of AI technologies making the detection of misinformation more difficult (Chiu, 2019) and spreading distrust: ‘It’s likely to spread distrust of all news on social media, further eroding public debate’ (Vaccari and Chadwick, 2020).

The example imaginaries also diverge regarding the envisioned implications, reflecting the different, and complex, cross-sectoral nature of the three imaginaries. The first imaginary portrayed regulators as responsible for legally clarifying proper use of facial-recognition technology, as emphasised, for instance, by the Clearview AI guest authors: ‘Clearview AI would welcome federal regulation that would unravel the tangle of sometimes inconsistent and unconstitutional state and local laws. Congress should authorize, with proper safeguards, use by law enforcement and national-security authorities’ (Abrams and Wolosky, 2021). The second imaginary included corporate actors themselves laying out ‘next steps’ for enhancing consumers everyday lives: ‘Google also said it would soon add phone-call capabilities to its Google Home smart speakers’ (Nicas, 2017). Finally, the third imaginary envisioned a complex set of responsible actors spanning private sector (social media platforms), public sector (policymakers), and civil society (citizens) to be prepared for and limit the spread of deepfakes: ‘politicians, social media platforms and citizens might want to prepare for the spread of deepfakes. In addition to limiting their spread online, policymakers may wish to take active measures to boost news consumers’ trust in good quality information’ (Vaccari and Chadwick, 2020).

But the three example imaginaries also share several similarities, from the futures dimension, to the related certainty assessments, to the speaker dimension.

Regarding the spatio-temporal futures dimension, the envisioned timespan for realising the imaginary was not specified for the first imaginary, and described in vague and contradictory terms for the second, as it ranged from ‘soon’ (Nicas, 2017) and ‘this fall’ (Mims, 2021) to ‘in the coming years’ (Baig, 2017). The timespan was only clearly described for the third imaginary – as already happening in the present, but expected to worsen in the future: ‘The technology behind fake videos is improving almost on a continuous basis’ (Guynn, 2020). The spatial element of the imaginary remained unspecified or without clear theme across all three imaginaries, with the lack of a clear theme in the crime-fighting imaginary being evident in mentions of varying spaces including ‘France’ (Meichtry and Schechner, 2021), ‘China’ (Harwell and Dou, 2020), and the ‘EU’ (Schechner and Olson, 2021).

Certainty assessments about realising the vision were similar for the second and third imaginary, which were presented as highly certain, whereas the first was presented ambiguously, including both portrayals of certainty and uncertainty. First, enhancing consumers’ everyday life as well as the manipulation of public perceptions is described as something that will happen and that is already happening, but getting worse, respectively. For consumers ‘you’ll be able to expand the Google Assistant’s presence, to order an Uber by voice, make coffee, turn on music or ask about the weather’ (Baig, 2017), while for public perception manipulation, one article even explicitly declared: ‘Fair warning: It’s going to get worse’ (Guynn, 2020). Fighting crime was described both with certainty due to first signs of success online, such as that ‘Artificial intelligence is already improving Facebook’s ability to stop the spread of terrorist content on Facebook’ (Guynn, 2017), as well as uncertainty regarding facial-recognition technology, also described as ‘ethnicity-detecting software’ in one article: ‘It’s less certain whether ethnicity-detecting software could ever take off outside the borders of a surveillance state’ (Harwell and Dou, 2020).

The speaker dimension shows the largest similarity between all three imaginaries. Technology sector and civil society sector representatives are proponents of all three imaginaries in U.S. news coverage. The only difference lies in the inclusion of government representation in the case of the more public sector-focused imaginary of fighting crime. This suggests that the technology and civil society sectors are successful in shaping several, diverging imaginaries of AI at the same time. Indeed, technology sector speakers include, for example, Facebook Chief AI Scientist Yann Lecun (Uberti, 2020), Google CEO Sundar Pichai (Nicas, 2017), and ‘Facebook’ (Vaccari and Chadwick, 2020) in the first, second and third imaginary, respectively. Civil society, on the other hand, includes speakers such as ‘privacy and digital-rights activists’ (Meichtry and Schechner, 2021) in the first imaginary, consumers through a global online survey (Baig, 2018) in the second, and an associate professor of computer science at the University of Southern California (Guynn, 2020) in the third. Speakers in the first, crime-fighting imaginary include, for example, French Prime Minister Jean Castex (Meichtry and Schechner, 2021).

In sum, these examples illustrate that SIPCs, in this case of AI, can vary considerably in their visions, specific technological object, desirability, and resulting implications, while still displaying similarities in other dimensions, such as the spatio-temporal futures and the certainty descriptions, and speaker representation. Furthermore, these examples illustrate the possible co-existence of different imaginaries in the same national context, media and points in time. However, while this illustration focuses on subtle similarities in very different examples, the framework's complexity could also allow for the identification of subtle differences in what may appear to be established, and largely similar, cross-national or cross-media imaginaries.

Overall, this exemplifies the usefulness and applicability of the analytical framework, and its ability to extract subtle varieties from empirical material. What the examples could not yet show is the dynamic interplay and potential competition between different imaginaries in public communication over time. Analysing such competitions for public visibility and interpretive hegemony in public communication, as proposed and partly done by scholarship on public communication of science and technology before, will be a helpful next step in assessing the usability of the SIPCs framework, and in gaining deeper insights into the imaginaries of complex, emerging technologies such as AI.

## Conclusion

Public communication contributes to the communicative construction of sociotechnical imaginaries. However, the way in which this communicative construction takes place has previously remained under-conceptualised. While the concept of SIs provides a framework to assess visions of sociotechnical futures in general, its operationalisation of the communicative dimension remained vague despite the explicit acknowledgement of the crucial role of public communication in the construction of SIs (Jasanoff, 2015a).

We consider SIs to be a valuable building block to understand the communicative construction of technologies due to the concept's ability to capture the mutual, iterative shaping between society and technology, its integration of discursive and institutional elements as well its orientation toward the future – while also perceiving the need to specify the communicative layer of the concept. We therefore combined this strand of research with scholarship on PCT, which has suggested that public and particularly mediated communication have the ability to profoundly influence the trajectories of technologies in society, by educating the public and influencing attitudes about these technologies (Bauer, 2005; Brossard, 2013). But so far, this research had refrained from systematically conceptualising how visions of sociotechnical futures may manifest in public communication and how they highlight trajectories for technological development. Accordingly, a mutual approximation of both approaches for the development of a rigorous analytical framework seemed worthwhile.

Therefore, we proposed the SIPCs framework to systematically capture visions of sociotechnical futures as they manifest themselves in public communication. Sociotechnical Imaginaries in Public Communication are oriented towards sociotechnical futures, aimed toward inspiring collective motivation for support or opposition, and connected to concrete actions for shaping those futures. As such, they are not only descriptive, but also prescriptive. From the SI concept we drew four **imaginary** dimensions: **visions**, **desirability, futures** and **object**. **Visions** contain an element defining the envisioned *role* of the technology, as well as one element regarding the *certainty* about the realisation of the vision. **Desirability** contains elements regarding the (un)desirability *evaluation* of the vision, the *affected entities*, and the *rationale* for the desirability judgement. The **futures** dimension contains the expected *timespan* and *geographical scale* expressed for the vision. The technological **object** entails both a specification of the *technology element* and of the *technology context*. From scholarship on public communication about technologies, we drew two additional dimensions that contextualise the core imaginary dimensions, namely the **speaker** and the **implications** dimensions. We suggest that the former – the **speaker** – is an element in and of itself that captures the person, organisation or other entity communicating and promoting the vision. The latter – **implications** – include an *action recommendation*, which delineate actions to be performed to promote or prevent the realisation of the imaginary, and an articulation of the *action responsibility*, which are the people, organisations or other actors deemed responsible for the implementation of the recommended actions. We drew three empirical illustrations of SIPCs of AI from news coverage in the United States. They showed, first, that even within a single national context, medium type and timespan, very different imaginaries can co-exist. They also showed that, through comparisons across the different dimensions and elements of the framework, subtle similarities and differences can come to the forefront, which may otherwise have been overlooked. The SIPCs framework lends itself to systematic empirical analyses – both in-depth qualitative and large-scale quantitative examinations – of stakeholder communication, news and social media representations and other forms of public communication about emerging and envisioned future technological advances.

Nevertheless, our proposal and its empirical illustration also engenders several implications and points of discussion regarding its future application:

First, we focused mainly on the *shape* that sociotechnical imaginaries take in public communication. Beyond that, it is important for imaginaries to garner collective support. This will allow it to carry enough weight in public perception in order to impact the materialisation of an imaginary: investments in realising the vision, regulation to advance or prevent the vision, furthering of skills and habits to realise or prevent the vision. Future research should investigate the *spread of SIPCs* to determine the tipping point at which an imaginary has garnered enough support, is being sufficiently ‘publicly performed’ (Jasanoff, 2015a) to warrant the status as fully stabilised.

Second, while the *time* and/or *space* in the publicly constructed visions of (un)desirable sociotechnical future*s* might not be explicitly laid out, one central aspect of an SIPC is that it is turned toward a future. In fact, even when public communication does not explicitly lay out a timespan for the vision, it may still contain a *temporal* element that clearly is *turned toward the future*. Markers for such a future-orientation could be the use of the future or conditional tense in promoting the vision, a promotion of what *can* happen if the vision for the technology is realised, or a description of a vision that is presented as something expanding, improving or worsening as time progresses.

Third, regarding the *object* dimension, in more stabilised technological cases, the *technology characteristics* may be less variable, whereas the *technology context* might vary – for instance, in the example of nuclear fission, the context could be both for power generation, as well as for nuclear weaponry. In other instances, there might be only one specific technology being referred to in only one specific context. But as we showed in the example we used for illustrating our concept in this paper, artificial intelligence, the still persisting interpretive flexibility of complex, emerging or developing technologies (Bijker, 2009) means that there can be a wide variety of both *technology characteristics* and *technology contexts*.

Fourth and finally, while the framework was primarily developed for semantic analysis of SIPCs in textual data, it can also inform structured semiotic analyses of sociotechnical imaginaries in visual, audio or multimodal public communication by enriching the analysis of particular elements with approaches from multimodal social semiotics (Kress and Mavers, 2005). While an in-depth proposal of how to approach such an analysis exceeds the scope of this article, we suggest a few starting points here. First, to identify *future-orientation*, researchers would need to rely on some numeric or textual signs marking the future, either in metadata (e.g. image description, film synopsis, etc) or within the visual, audio or multimodal object. Similarly, speakers could be identified in metadata (e.g. author, photographer, etc) or as cited speakers, marked, for example, through speech bubbles in images or banners or spoken announcements in audio (visual) data. Drawing again on the example of AI and depending on the analytical goal, an analysis of the *technological object* could, for instance, explore the level of anthropomorphisation of AI in image and sound (Barrow, 2024), looking for humanlike or more abstract features of AI representations in images or mechanic sounds versus the presence or absence of a more-or-less humanlike voice in audio. An analysis of the *technological context* could identify visual or sonoric signs (Kress and Mavers, 2005) representing different societal sectors, like healthcare, media, education, or industry. Using again the example of AI, an analysis of the *role* could, for example, explore the envisioned role of AI in relation to humans (Scott Hansen, 2022) by analysing the position, size, or shape of the AI and the human(s) in an image (Kress and Mavers, 2005). While somewhat less clear-cut, analyses of the *desirability level* and vision *certainty* in images could draw from established grammar of colour (Kress and Van Leeuwen, 2002), referring to grey-scale value (light to dark) or colour saturation (saturated to pale) for the former, and colour modulation (fully modulated to flat) for the latter, potentially combined with hue scale value (blue to red) for either. The *space*, if specified, could be inferred from visual or sonoric signs (Kress and Mavers, 2005), like references to local or national music and sounds or landmarks. Similarly to the futures element, the *treatment recommendations* and *responsibility* elements would be more reliant on text than some of the other elements. While the discussion here has remained short, we hope that it will provide a fruitful starting point for analysing the construction of SIs in non-textual or multimodal public communication.

## Data Availability

The data that support the findings of this study are available from Factiva database but restrictions apply to the availability of these data, which were used under license for the current study, and so are not publicly available. Data are however available from the authors upon reasonable request and with permission of Factiva database.*

## Note

1. Later checks revealed that this preliminary U.S. corpus also included 43 articles from 2010 to 2011 and 6 articles from the *New Yorker*, and was missing 135 articles. False positives were excluded from final analysis. False negatives displayed a random distribution between 2014 and 2021 and across outlets and should not considerably affect the final analysis and results.
